# Suppression of Pancreatin-Induced Digestion of Starch in Starch Granules by Starch/Fatty Acid and Starch/Flavonoid Complexes in Retrograding Rice Flour

**DOI:** 10.3390/foods7080128

**Published:** 2018-08-10

**Authors:** Sachiko Hirota, Umeo Takahama

**Affiliations:** Department of Health and Nutrition Care, Faculty of Allied Health Sciences, University of East Asia, Shimonoseki 751-8503, Japan; hirotasa@toua-u.ac.jp

**Keywords:** adzuki bean (*Vigna angularis*), mechanisms of starch digestion suppression, retrograding non-glutinous rice (joshinko), starch/fatty acid complexes, starch/flavonoid complexes

## Abstract

Adzuki beans are used to prepare foods with glutinous and non-glutinous rice in Japan, and adzuki bean pigments are able to color rice starch a purplish red. This study deals with the adzuki bean extract-dependent suppression of starch digestion of non-glutinous rice flour (joshinko in Japanese), which was gelatinized in boiling water and then cooled to 37 °C. Accompanying the treatment of joshinko with pancreatin, amylose and amylopectin were released from the joshinko particles, and the released amylose and amylopectin were further digested. The adzuki extract suppressed the release and digestion by binding to amylose and amylopectin, which were present in the particles and at the surfaces of the particles. Fatty acids and flavonoids in the adzuki extract contributed to the suppression. In addition, the starch digestion in the joshinko particles appeared to be suppressed if the amylose/fatty acid complexes and amylose/flavonoid and amylopectin/flavonoid complexes, which are poor substrates of α-amylase, surrounded the particles. It is discussed that the suppression was due to the prevention of α-amylase access to the particles.

## 1. Introduction

Adzuki bean is a normal food in Japan and is used in cooking with glutinous and non-glutinous rice to prepare sekihan and adzuki-meshi, respectively. The adzuki bean is also used to prepare the paste for sweets. In addition, flour of non-glutinous rice (joshinko in Japanese) is normally used to prepare various Japanese foods, including dumplings, uiroh (sweet jelly or pudding), kashiwa-mochi (rice cake wrapped with an oak leaf), kusa-mochi (rice cake mixed with boiled leaves of mugwort), etc. The latter two types of cakes are filled with adzuki bean paste. 

It has been reported that adzuki contains health-promoting antioxidants, such as flavonoids and tocopherols [1,2,3], and that the flavonoids are transferred to the rice when cooked with rice [4]. Recently, we have reported that a diethyl ether-extract of the adzuki bean can suppress starch digestion of the homogenates of cooked non-glutinous rice and that taxifolin, quercetin, and a purplish pigment vignacyanidin (a cyanidin-catechin conjugate) in the extract can contribute to the suppression [5,6]. The efficiency of the suppression increases with the increase in hydrophobicity of the flavonoids, namely, taxifolin < quercetin < vignacyanidin, but it is difficult to distinguish the effects of the adzuki extract on the digestion between amylose and amylopectin [5,6,7]. However, when joshinko was used instead of homogenized rice, we noticed that it was possible to distinguish the digestion of amylose from amylopectin by measuring the absorption spectra of starch-iodine complexes. 

This study deals with (i) the adzuki bean extract-dependent suppression of starch digestion in retrograding joshinko, which was catalyzed by α-amylase in pancreatin; (ii) the binding of adzuki components to amylose and amylopectin in retrograding joshinko; and (iii) the elucidation of the adzuki bean components that suppressed starch digestion. Taking the above results and the structures of retrograding joshinko particles into consideration, it is discussed that amylose/fatty acid complexes and amylose/flavonoid and amylopectin/flavonoid complexes can suppress starch digestion in the particles by surrounding them. The elucidation of the mechanism may provide some procedures to prepare foods using joshinko, the digestion of which is slow. 

## 2. Materials and Methods

### 2.1. Ingredients and Reagents

Seeds of adzuki (*Vigna angularis* (Willd.) Ohwi & H. Ohashi cv. erimo) were supplied by the Japan Pulse Foundation (Tokyo, Japan) and the Tokachi Agricultural Experiment Station (Tokachi, Japan). Flour of non-glutinous rice (joshinko) was obtained from Hinode-Seifun Co. (Kumamoto, Japan). (+)-Catechin, taxifolin, rutin, and quercetin were from Sigma-Aldrich Japan (Tokyo, Japan). Oleic acid, linoleic acid, linolenic acid, and pancreatin from pig pancreas were from Wako Pure Chemical Ind. Co. (Osaka, Japan). Quercetin 7-*O*-glucoside and vignacyanidin were prepared from adzuki bean as reported previously [8,9].

### 2.2. Preparation of Adzuki Extract

Two liters of water was added to 1 kg of dried adzuki bean and kept at 25 °C in the dark for about 15 h. Methanol (2 L) was added to the soaked adzuki bean after removing the water not absorbed by the bean. The adzuki bean in methanol was kept at 25 °C in the dark for 1 h with gentle shaking to extract components contained in the seed coat. This extraction was repeated twice. After combining the two methanol extracts, methanol was removed in vacuo and the pH of the remaining aqueous solution (about 700 mL) was adjusted to 3 by HCl to extract with 200 mL of ethyl acetate three times. Water not absorbed by the adzuki bean (about 500 mL) was extracted with 200 mL of ethyl acetate once after the pH was decreased to 3. The ethyl acetate extracts were combined and washed with 100 mL of 1% HCl five times. After removing the water of the combined ethyl acetate extracts with anhydrous sodium sulfate, ethyl acetate was evaporated in vacuo. The residue was dissolved in 2 mL of ethanol with 0.1% HCl and kept at −20 °C. Flavonoids in the extract were identified from their retention times, UV-visible absorption spectra, and mass spectra [4]; their amounts were calculated from the areas under the peaks.

### 2.3. Preparation of Retrograding Joshinko

Joshinko (0.5 g) was suspended in 50 mL of 0.1 M sodium phosphate (pH 7.0) with 0.15 M NaCl, and gently heated for 5–6 min to start boiling. After gentle boiling for 2 min, the gelatinized joshinko was cooled to 37 °C in a water bath. The cooled joshinko was used for experiments as retrograding joshinko. Adzuki extract (0–10 μL) was added to 1 mL of the retrograding joshinko (0.01 g mL^−1^) and then incubated for 5 min at 37 °C. The reason that the volumes were selected was that 10 μL of adzuki extract, which was equivalent to 5 g of adzuki bean, significantly suppressed pancreatin-induced starch digestion under the conditions of this study. Retrograding joshinko with and without adzuki extract was centrifuged at about 1500× *g* for 2 min. The supernatants and sediments were reddish purple and purple, respectively. The purple sediments were washed once with 1 mL of the above buffer solution and then suspended in 0.9 mL of the buffer solution. In the following experiments, the samples used were (1) retrograding joshinko, (2) the sediment, and (3) the supernatant. Starch digestion was initiated by adding 5 or 10 μg of pancreatin mL^−1^ at 37 °C. 

### 2.4. Estimation of Starch Digestion

Starch digestion was estimated by the reducing sugar formation and starch-iodine complex formation. The solution to quantify reducing sugars was prepared by mixing solutions I and II (1:9, *v*/*v*) just before the experiments [10]. Solution I contained 0.33 M 4-hydroxybenzhydrazide and 0.6 M HCl; solution II contained 0.042 M sodium citrate, 0.007 M CaCl_2_, and 0.5 M NaOH. Samples (1)–(3) were incubated for defined periods with pancreatin, and 20 μL of the samples was added to the mixture of the two solutions and kept in boiling water for 6 min. After cooling in iced water and centrifuging at 11,000× *g* for 2 min, absorbance of the supernatant was measured at 410 nm using a spectrophotometer (UV-2550, Shimadzu, Kyoto, Japan). The path length of the measuring beam was 4 mm.

Iodine solution (100 mM) was prepared as described previously [7]. After incubating samples (1)–(3) for defined periods with pancreatin (10 μg mL^−1^), 30 μL of the reaction mixture was withdrawn and added to the mixture of 10 mM iodine in 1.0 mL of 0.1 M sodium phosphate (pH 7.0) with 0.15 M NaCl. As large joshinko particles were precipitated rapidly after the addition of the reaction mixtures prepared from samples (1) and (2), i.e., joshinko and the sediment, the absorption spectra of starch-iodine complex were recorded 1 min after the addition of the reaction mixtures using a UV-2450 spectrophotometer combined with an integrating sphere (MPS-2000) (Shimadzu). The path length of the measuring beam was 4 mm. Data are presented as the difference in absorbance between the starch-iodine complexes and the iodine solution at 550 and 700 nm.

### 2.5. Measurements of Total Sugar

Total sugar was estimated by the phenol-sulfuric acid method [11]. An aliquot (20 μL) of the reaction mixture was added to 1 mL of 5% phenol solution, and then 1 mL of water was added. After adding 5 mL of concentrated sulfuric acid to the 2 mL phenol solution, the mixture was left for 10 min and cooled to room temperature in a water bath. The cooled solution was centrifuged at about 1500× *g* for 5 min, and then the absorbance of the supernatant was measured at 490 nm. The concentrations of total sugar were estimated from the standard curve prepared using glucose, and the total sugar concentrations were expressed equivalent to mg or μmol of glucose. 

### 2.6. Estimation of Flavonoid and Fatty Acid Contents by HPLC

One milliliter of retrograding joshinko (0.01 g mL^−1^) incubated with 10 μL of adzuki extract for 5 min at 37 °C was centrifuged at about 1500× *g* for 2 min. The sediment was washed once with 1 mL of 0.1 M sodium phosphate with 0.15 M NaCl (pH 7), and then the sediment was extracted with 10 mL of 0.1% HCl in methanol. The supernatants obtained by the first and the second centrifugation were combined and then extracted with 10 mL of 0.1% HCl in methanol. The methanol extracts were dried in vacuo using a rotary evaporator. Each residue was dissolved in 0.5 mL of 1% HCl in methanol, and an aliquot (50 μL) of each solution was applied to a HPLC column (Shim-pack VP-ODS; 150 mm × 4.6 mm i.d.) combined with a pump (CL-20AD) and a photodiode array detector (SPD-M20A) (Shimadzu, Kyoto, Japan). The mobile phase was a mixture of methanol and 0.2% formic acid and the concentration of methanol increased stepwise as previously reported [4,6]. The flow rate of each mobile phase was 1 mL min^−1^. Each flavonoid was quantified from the area under the peak.

Fatty acids in adzuki extract were also estimated using the above HPLC system. The mobile phase was a mixture of methanol and 0.2% formic acid (9:1, *v*/*v*), and the flow rate was 1 mL min^−1^. Fatty acids separated were detected at 210 nm and quantified from the area under the peak.

### 2.7. Reconstitution Experiments

About 0.8 mL of the supernatant was obtained by the centrifugation of 1 mL of joshinko (0.01 g mL^−1^) at 1500× *g* for 5 min. The supernatant, which might have contained starch fragments, was incubated with the adzuki extract, fatty acid, and flavonoid for 5 min at 37 °C. The supernatant became turbid by the incubation. Then, the turbid mixtures were centrifuged at 11,000× *g* for 5 min to obtain the precipitates. The precipitates were suspended in 0.8 mL of 0.1 M sodium phosphate (pH 7.0) with 0.15 M NaCl and mixed with the sediment obtained by the centrifugation of retrograding joshinko at 1500× *g* to study starch digestion induced by 10 μg of pancreatin mL^−1^. The digestion was estimated using iodine as described above.

### 2.8. Presentation of Data

Measurements of pancreatin-induced starch digestion were repeated three times using different joshinko preparations. Quantification of flavonoids and fatty acids in the adzuki extract was also repeated three times using the adzuki bean extract that was used throughout this study. In principle, data are presented as means with SDs.

## 3. Results and Discussion

### 3.1. Starch Digestion Estimated by Reducing Sugar Formation

**Figure 1:**
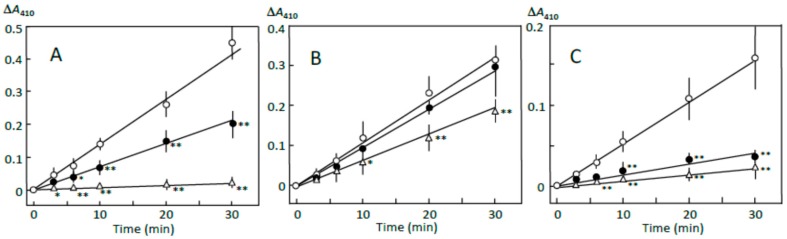
Adzuki extract-dependent inhibition of reducing sugar formation. The reaction mixture contained (**A**) retrograding joshinko, (**B**) the sediment, and (**C**) the supernatant in 0.1 M sodium phosphate (pH 7.0) with 0.15 M NaCl. Reactions were initiated by adding (**A**) and (**B**) 10 μg mL^−1^ or (**C**) 5 μg mL^−1^ of pancreatin. (○) no addition, (●) 5 μL, (∆) 10 μL of the adzuki extract. Each data point is an average with SD (*n* = 3). * *p* < 0.05; ** *p* < 0.01.

The pancreatin-induced reducing sugar formation in retrograding joshinko was suppressed by 5 and 10 μL of adzuki extract by about 50% and more than 95%, respectively (Figure 1A). Rates of the reducing sugar formation in the sediment prepared with 5 and 10 μL of adzuki extract as described in Section 2.3 were about 90% and 40%, respectively, of that in the sediment prepared without the adzuki extract (Figure 1B). On the other hand, the rates of the supernatant prepared in the presence of 5 and 10 μL of adzuki extract were about 20% and 10%, respectively, of that prepared in the absence of the adzuki extract (Figure 1C). The quite slow reducing sugar formation was not due to the adzuki extract-dependent decrease in the starch concentrations. This was supported by the results, which showed the amounts of total sugar in the supernatant were equivalent to 2.58 ± 0.02, 1.85 ± 0.33*, and 1.46 ± 0.03* mg of glucose (*n* = 3; * *p* < 0.05) when the supernatant was prepared in the presence of 0, 5, and 10 μL of adzuki extract, respectively. The results in Figure 1 suggest that the components in the adzuki extract could control starch digestion by interacting with amylose and/or amylopectin fragments, which could be separated from the joshinko particles by centrifugation. To further discuss the mechanisms of the suppression, starch digestion was studied using iodine.

### 3.2. Starch-Iodine Complex Formation in Retrograding Joshinko

**Figure 2:**
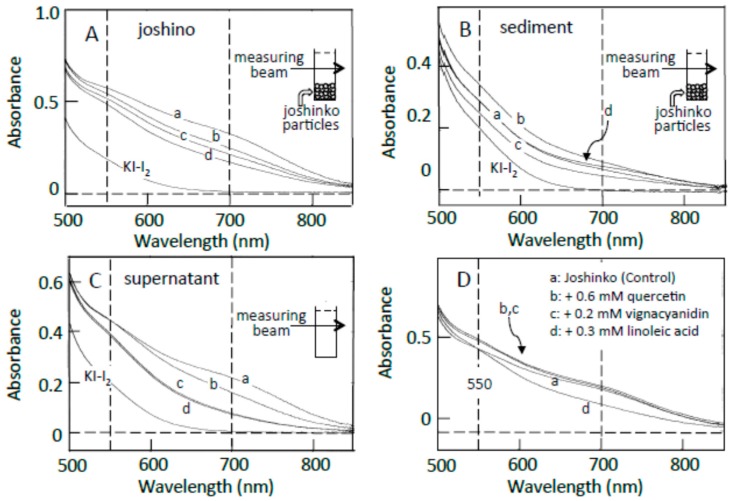
Effects of adzuki extract on the formation of starch-iodine complexes. (**A**) retrograding joshinko, (**B**) the sediment, (**C**) the supernatant. KI–I_2_: iodine solution. (**a**) 0 μL, (**b**) 3 μL, (**c**) 6 μL, (**d**) 10 μL of the adzuki extract. Absorption spectra of the starch-iodine complexes were measured after the precipitation of joshinko particles as illustrated in each panel. (**D**) Effects of 0.6 mM quercetin, 0.2 mM vignacyanidin, and 0.3 mM linoleic acid. Here, the absorption spectra were also measured after the precipitation of joshinko particles.

**Figure 3:**
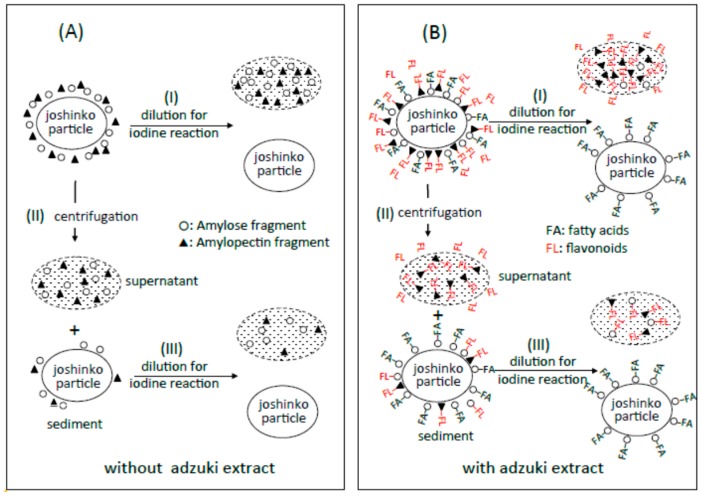
Possible schemes for the arrangement of amylose (○) and amylopectin (▲) around joshinko particles in retrograding joshinko. (**A**) Without adzuki extract and (**B**) with 10 μL of the adzuki extract. For details, see text.

Traces a in Figure 2A–C show the absorption spectra of the starch-iodine complexes of retrograding joshinko, the sediment, and the supernatant, respectively, which were recorded after the large joshinko particles had precipitated in the cuvette (see Materials and Methods). Two shoulders around 550 and 700 nm in the joshinko (A) and the supernatant (C) suggest the release of loosely bound amylose and amylopectin from the particles in the retrograding joshinko by the dilution to measure starch-iodine complex formation (I) and by centrifugation (II), respectively (Figure 3A). The joshinko used in this study was prepared by milling non-glutinous rice. Therefore, the presence of fragmented amylose and amylopectin in the flour is possible. In addition to these fragments, amylose in joshinko particles is supposed to move to the surface of starch granules present in the particles during gelatinization [12,13,14]. The ∆*A* spectrum of trace a minus KI–I_2_ in Figure 2B had a broad peak around 600 nm (not shown), indicating that amylopectin and amylose precipitated with joshinko particles by centrifugation were released from the sediment when suspended in the buffer solution to measure the starch-iodine complex formation (III).

The adzuki extract suppressed the amylose–iodine complex formation, which had an absorption maximum around 700 nm, in retrograding joshinko (Figure 2A). According to this result, the adzuki extract-dependent decrease in amylose concentration in the supernatant (Figure 2C) was most likely due to the precipitation of amylose, which was combined with the adzuki components, by centrifugation. The absorption spectra of amylose and amylopectin released from the sediments, which were prepared in the presence of the adzuki extract (traces c–d), were not so different from that of trace a (Figure 2B), suggesting that the adzuki components did not significantly affect their characteristics to combine with iodine.

As the components to suppress the starch-iodine complex formation in retrograding joshinko, fatty acids were considered first as adzuki beans contain fatty acids [15], which could suppress starch-iodine complex formation [16,17,18,19] and form precipitable amylose/fatty acid complexes [20,21,22]. Oleic (C18:1), linoleic (C18:2), and linolenic (C18:3) acids were identified in the adzuki extract by the HPLC system described in Materials and Methods, and their concentrations in the extract were estimated to be 71.1 ± 12.8, 4.8 ± 1.4, and 3.6 ± 0.6 mM (*n* = 3), respectively. Here, the concentration of oleic acid was much higher than that of linoleic acid, but it has been reported that the content of linoleic acid is greater than that of oleic acid in the adzuki bean [23,24]. Therefore, both oleic and linoleic acids were used here. Linoleic acid suppressed amylose–iodine complex formation (Figure 2D, trace d), and the suppressive effects were nearly saturated at 0.3 mM (data not shown). Essentially the same result was obtained when oleic acid was used. The results suggest that fatty acids in the adzuki extract could make amylose precipitable, preventing the formation of amylose–iodine complexes. The concentration of oleic acid was estimated to be about 0.7 mM when 10 μL of adzuki extract was added.

In addition to fatty acids, flavonoids can suppress starch iodine complex formation [7,25,26] because of their presence in the adzuki extract [4,6]. Table 1 shows flavonoid concentrations in the adzuki extract used in this study. In the flavonoids, quercetin and vignacyanidn could combine with joshinko particles more effectively than other flavonoids. It has been reported that these flavonoids can bind to starch effectively [5,6]. Figure 2D (traces b and c) shows the effects of 0.6 mM quercetin and 0.2 mM vignacyanidin (the concentrations of which were higher than those in the presence of 10 μL of adzuki extract) on the starch-iodine complex formation using retrograding joshinko. During the incubation of the joshinko with quercetin or vignacyanidin for 5 min, intense yellow or purple joshinko particles precipitated and the colors of the supernatants were faint yellow and purple, respectively. Although these flavonoids were present in the supernatants, they did not prevent starch-iodine complex formation under the conditions of this study.

From the effects of the adzuki extract on starch-iodine complex formation, it could be postulated (Figure 3B) that (i) the dilution of the adzuki extract-containing joshinko resulted in the separation of joshinko particles and amylose/fatty acid complexes from the amylose and amylopectin combined with flavonoids (I); (ii) the centrifugation of the joshinko also resulted in the precipitation of joshinko particles and amylose/fatty acid and amylose/flavonoid complexes, leaving amylopectin combined with flavonoids in the supernatant (II); and (iii) amylopectin and amylose, which coprecipitated with joshinko particles, were released from the particles when suspended in the buffer solution to measure starch-iodine complex formation (III).

### 3.3. Starch Digestion of Retrograding Joshinko Estimated Using Iodine

**Figure 4:**
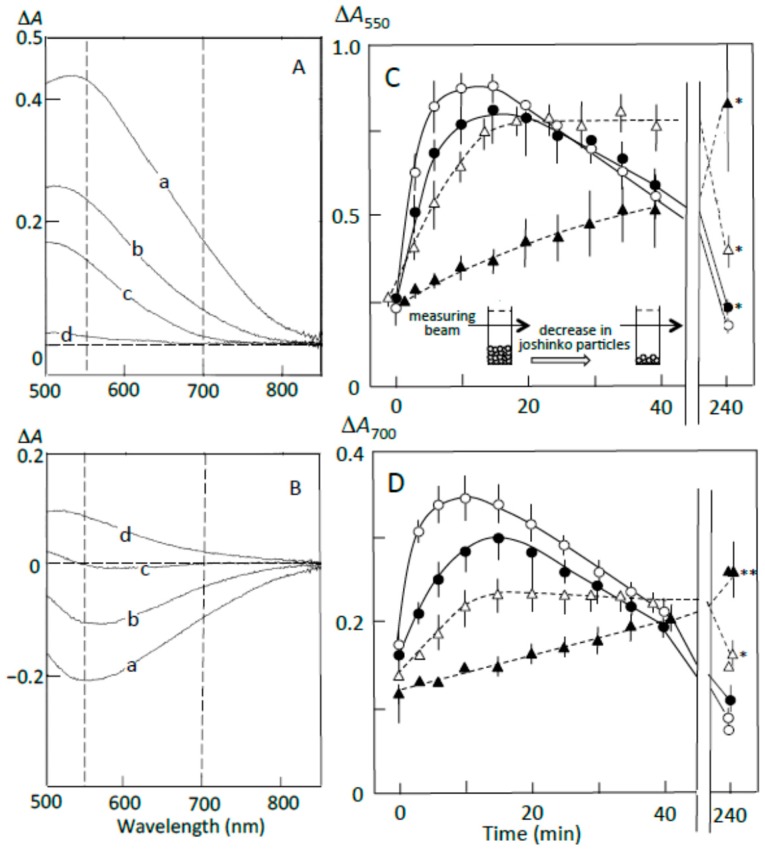
Pancreatin-induced starch digestion of retrograding joshinko. (**A**,**B**) ∆*A* spectra of 3 min minus 0 min and 40 min minus 20 min of incubation, respectively. (**a**) 0 μL, (**b**) 3 μL, (**c**) 6 μL, (**d**) 10 μL of the adzuki extract. (**C**,**D**) Time courses of starch release and the digestion of released starch measured at 550 and 700 nm, respectively. (○) 0 μL, (●) 3 μL, (∆) 6 μL, (▲) 10 μL of the adzuki extract. Each data point is the average with SD (*n* = 3). * *p* < 0.05; ** *p* < 0.01.

Figure 4A shows the pancreatin-induced release of starch fragments from joshinko particles. The ∆*A* spectrum of 3 min minus 0 min recorded in the absence of the adzuki extract had a peak of absorbance increase around 550 nm, with a considerable absorbance increase around 700 nm (trace a). This result suggests the release of not only amylopectin but also amylose from the joshinko particles. The adzuki extract suppressed the absorbance increase around 700 nm more effectively than that around 550 nm (traces b–d). This was confirmed by the time courses of the initial increase in the starch concentrations (Figure 4C,D). These results suggest that the adzuki extract suppressed the release of amylose more effectively than that of amylopectin.

The ∆*A* spectra of 40 min minus 20 min of incubation in the presence of pancreatin are shown in Figure 4B. The spectrum in the absence of the adzuki extract had a peak of absorbance decrease around 550 nm, with a considerable absorbance decrease around 700 nm (trace a), suggesting the digestion of released amylose and amylopectin during the incubation period. The adzuki extract (3 and 6 μL) suppressed the digestion of the released amylose and amylopectin (traces b and c). In trace d, the ∆*A* spectrum had a positive peak around 550 nm and the ∆*A* at 550 nm was much greater than that at 700 nm, suggesting that amylopectin was mainly released from the joshinko particles during the incubation period in the presence of 10 μL of adzuki extract. The results in Figure 4B were confirmed by the time courses of starch concentration changes during the incubation period from 20 to 40 min in the presence and absence of the adzuki extract (Figure 4C,D).

The suppression of the released starch digestion by 6 μL of adzuki extract seemed to continue for 4 h, and the release of amylopectin in the presence of 10 μL of adzuki extract also seemed to continue for 4 h (Figure 4C,D). Joshinko particles that precipitated in the cuvette were still present after incubation for 4 h. Then, the packed volumes before and after the incubation were determined by centrifugation at 11,000× *g* for 2 min; the volumes decreased to 16 ± 1, 30 ± 7*, and 77 ± 17%* (*n* = 3; * *p* < 0.05) by incubation in the presence of 0, 5, and 10 μL of adzuki extract, respectively. The above results coincided with the observation that adzuki extract could suppress the release of starch fragments from joshinko particles and the digestion of the released fragments. 

According to the illustration that starch fragments surround joshinko particles in Figure 3, it is possible to postulate that (a) the adzuki extract-dependent suppression of amylose release from joshinko particles was due to the formation of amylose/fatty acid complexes in the particles and at the surface of the particles; and (b) the suppression of the initial release of amylopectin was due to the surrounding of joshinko particles by amylose/fatty acid complexes and by amylose/flavonoid and amylopectin/flavonoid complexes, which might be poor substrates for α-amylase [5,6,27,28,29]. In addition, the suppression of the released starch digestion by the adzuki extract might be due to the release of amylose/fatty acid complexes and the release of amylose and amylopectin combined with flavonoids. The following sections deal with the experiments to verify the above postulations.

### 3.4. Starch Digestion of the Sediment Estimated Using Iodine

**Figure 5:**
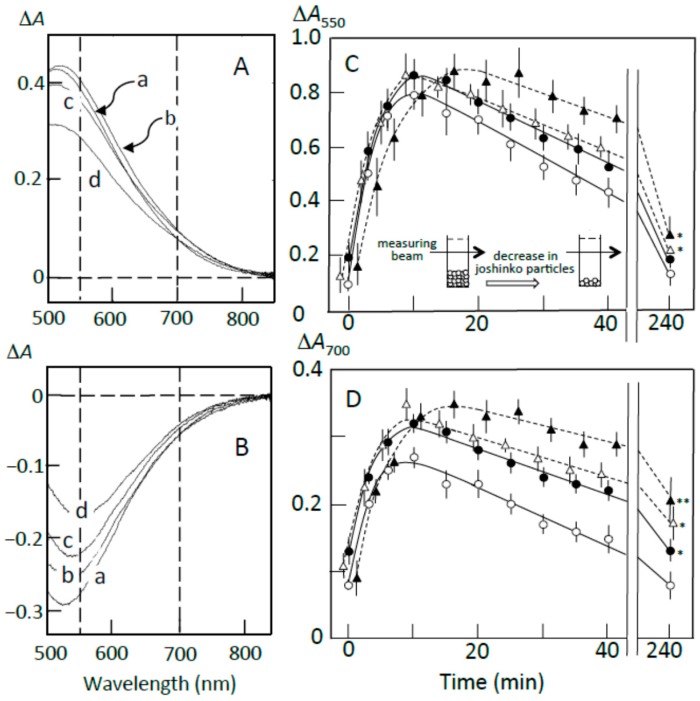
Pancreatin-induced starch digestion of the sediment. The sediment was prepared as described in Materials and Methods. (**A**,**B**) ∆*A* spectra of 3 min minus 0 min and 40 min minus 20 min of incubation, respectively. (**a**) 0 μL, (**b**) 3 μL, (**c**) 6 μL, (**d**) 10 μL of the adzuki extract. (**C**,**D**) Time course of starch release from the sediment and digestion of the released starch measured at 550 and 700 nm, respectively. (○) 0 μL, (●) 3 μL, (∆) 6 μL, (▲) 10 μL of the adzuki extract. Each data point is average with SD (*n* = 3). * *p* < 0.05; ** *p* < 0.01.

The pancreatin-induced release of starch fragments from the sediment is shown in Figure 5A. The ∆*A* spectrum of 3 min minus 0 min of incubation in the absence of adzuki extract showed a much greater absorbance increase around 550 nm than around 700 nm (trace a), suggesting that amylopectin was mainly released from the sediment. The adzuki extract suppressed the release, but the suppressive effects were smaller than those in Figure 4 (trace b–d). This was confirmed by the time courses of the release from the sediment (Figure 5C,D). The smaller suppression may be explained if one postulates that the amount of amylopectin combined with the adzuki components was smaller than that of amylopectin not combined with these components in the joshinko particles of the sediment. 

The ∆*A* spectra of 40 min minus 20 min of incubation (Figure 5B) indicate that the released amylopectin was digested, and the digestion was partly suppressed by the adzuki extract. From the time courses of the decrease in the starch-iodine complex concentration (Figure 5C,D), the adzuki extract was calculated to be able to suppress the digestion by about 10–40% depending on the amounts of adzuki extract added. The smaller suppression was supposed to be due to the release of amylopectin not bound with adzuki components. The suppression by the adzuki extract continued for 4 h, and the suppressive effects were greater at 700 nm than 550 nm. This result may suggest that amylose/fatty acid complexes, which are poor substrates of α-amylase [27,28,29], are included in the released components. The packed volumes after incubation for 4 h were 21 ± 2, 28 ± 4, 33 ± 3*, and 40 ± 4%* (*n* = 3; * *p* < 0.05) of the initial volumes in the presence of 0, 3, 6, and 10 μL of the adzuki extract, respectively. The above data support the postulation that the amylose and amylopectin combined with adzuki components had important functions to suppress the initial release of amylopectin from joshinko particles. 

### 3.5. Starch Digestion of the Supernatant Estimated Using Iodine

**Figure 6:**
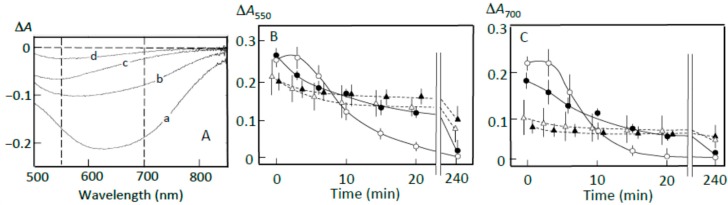
Pancreatin-induced starch digestion of the supernatant. (**A**) ∆*A* spectra of 10 min minus 0 min of incubation. (**a**) 0 μL, (**b**) 3 μL, (**c**) 6 μL, (**d**) 10 μL of adzuki extract. (**B**,**C**) Time courses of starch digestion estimated at 550 and 700 nm, respectively. (○) 0 μL, (●) 3 μL, (∆) 6 μL, (▲) 10 μL of the adzuki extract.

The ∆*A* spectra of pancreatin-induced digestion (Figure 6A) indicate that amylose was preferentially digested in the supernatant prepared in the absence of the adzuki extract (trace a) and that in the supernatants prepared in the presence of 6 and 10 μL of the adzuki extract, amylopectin was mainly digested (traces c and d). The digestion of amylopectin may be due to the greater decrease in amylose concentration relative to amylopectin concentration during the preparation of the supernatant in the presence of adzuki extract (Figure 2C). Time courses of the starch digestion showed that the adzuki extract could make amylose and amylopectin not easily digestible in the supernatant (Figure 6B,C). The above results suggest that if the not easily digestible amylose and amylopectin surrounded the joshinko particles, starch digestion in the particles might be suppressed. There is a possibility, however, that the suppression was due to the inhibition of α-amylase activity in pancreatin by free flavonoids, which might be present in the supernatant [30,31]. 

### 3.6. Suppression of Starch Digestion by Amylose and Amylopectin Combined with Adzuki Components

To exclude the above possibility that the suppression of starch digestion was due to the inhibition of α-amylase in pancreatin, the precipitate, which contained amylose and amylopectin combined with adzuki components, was prepared from the supernatant of retrograding joshinko as described in Section 2.7. The amounts of total sugar in the precipitates prepared by adding 1 and 5 μL of adzuki extract were equivalent to 1.7 ± 0.3 and 5.0 ± 0.5 μmol of glucose (*n* = 3), respectively, and the amounts of (+)-catechin, taxifolin, quercetin 7-*O*-glucoside, rutin, quercetin, and vignacyanidins in the blue precipitate prepared by adding 5 μL of adzuki extract were 2.20 ± 0.20, 1.59 ± 0.19, 1.61 ± 0.24, 0.27 ± 0.05, 18.7 ± 2.23, and 5.53 ± 0.67 (*n* = 3) nmol, respectively. The above data suggest that amylose and amylopectin in the supernatant became precipitable by combining with the components in the adzuki bean.

**Figure 7:**
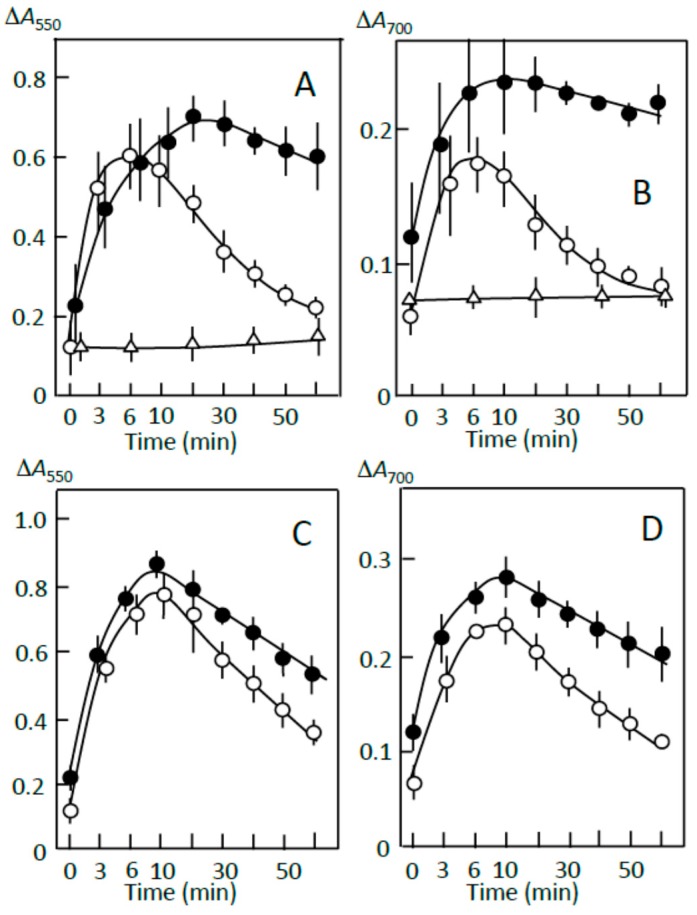
Starch digestion suppression by precipitates prepared using (**A**,**B**) adzuki extract and (**C**,**D**) fatty acid plus flavonoids. (○) sediment prepared from retrograding joshinko, (∆) precipitate prepared by adding 5 μL of adzuki extract or 0.6 mM oleic acid plus 0.6 mM quercetin to the supernatant, (●) sediment plus precipitate. For details, see Section 2.7. Each data point is the average with SD (*n* = 3).

Figure 7A,B show the effects of the above precipitate, which was prepared using 5 μL of the adzuki extract, on the digestion of the sediment prepared from retrograding joshinko in the absence of the adzuki extract. Amylopectin was mainly digested in the sediment, as shown in Figure 5C,D (○), and no significant starch digestion was observed in the precipitate prepared from the supernatant (∆). The latter result indicates that the adzuki extract could suppress starch digestion without inhibiting α-amylase activity as almost all of the adzuki components were bound to starch in the precipitate. The precipitate suppressed the initial release of starch, mainly amylopectin, from the sediment by 30–40% and suppressed digestion of the released starch by about 70% (●). The suppression was not due to the increase in concentration of not easily digestible starch in the reaction mixture because the amount of the precipitate added was less than 20% of that for the sediment under the conditions of Figure 7A,B. These results support the postulation that amylose and amylopectin combined with adzuki components could suppress the release of amylopectin from the sediment and the digestion of the released amylopectin. Therefore, it is possible that the mechanism of suppression involves the surrounding of joshinko particles and the released amylopectin by amylose and amylopectin combined with adzuki components. By the surrounding, the access of α-amylase to the joshinko particles and the released amylopectin can be prevented. 

### 3.7. Suppression of Starch Digestion by Fatty Acids and Flavonoids in Adzuki Extract

**Figure 8:**
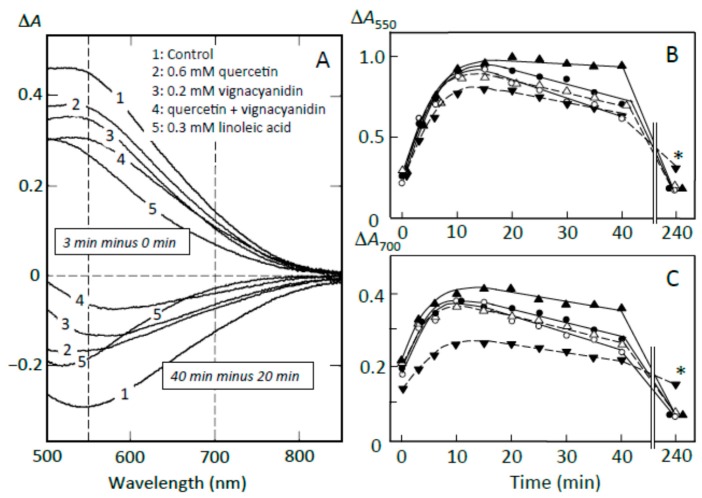
Effects of adzuki components on starch digestion of retrograding joshinko. (**A**) ∆*A* spectra. **Upper traces**, 3 min minus 0 min of incubation; **lower traces**, 40 min minus 20 min incubation. (**1**) no addition, (**2**) 0.6 mM quercetin, (**3**) 0.2 mM vignacyanidin, (**4**) 0.6 mM quercetin + 0.2 mM vignacyanidin, (**5**) 0.3 mM linoleic acid. (**B**,**C**) Time course of starch digestion measured at 550 and 700 nm, respectively. (○) no addition, (●) 0.6 mM quercetin, (∆) 0.2 mM vignacyanidin, (▲) 0.6 mM quercetin + 0.2 mM vignacyanidin, (▼) 0.3 mM linoleic acid. (○, ●, ▼) average of three experiments, (▲) average of two experiments. * *p* < 0.05.

To clarify the components that contributed to the suppression of starch digestion, the effects of fatty acids, quercetin, and vignacyanidin on pancreatin-induced starch digestion were studied using retrograding joshinko. There have been reports on the suppression of amylose digestion by fatty acids [27,28,29] and on quercetin- and vignacyanidin-dependent suppression of the digestion of amylose and amylopectin [5,6,26,32,33]. Figure 8A (upper traces) shows that the pancreatin-induced increase in starch-iodine complex formation (trace 1) was suppressed by flavonoids nearly equally at 550 and 700 nm (traces 2–4), whereas the formation was suppressed by fatty acids more significantly at 700 nm than 550 nm (trace 5). These data suggest that linoleic (or oleic) acid suppressed the release of amylose more effectively than that of amylopectin, whereas the flavonoids seemed to suppress the release of amylose and amylopectin equally. From the time courses at 550 and 700 nm (Figure 8B), it was calculated that 0.6 mM quercetin, 0.2 mM vignacyanidin, and 0.3 mM linoleic (or oleic) acid suppressed the release of starch by 16.0 ± 5.3, 22.2 ± 4.6, and 43.3 ± 10.5 % (*n* = 3), respectively, at 550 nm, and 13.7 ± 10.3, 22.1 ± 6.5, and 62.1 ± 15.1% (*n* = 3), respectively, at 700 nm. The suppressions by 0.6 mM quercetin plus 0.2 mM vignacyanidin were about 31 and 16% (average of two experiments) at 550 and 700 nm, respectively. 

Furthermore, the above concentrations of quercetin, vignacyanidin, and fatty acids suppressed the digestion of released starch fragments observed between 20 and 40 min (Figure 8A, lower spectra). Again, the fatty acids suppressed the digestion of amylose more effectively, and the flavonoids suppressed the digestion of amylose and amylopectin equally. Degrees of the suppression by 0.6 mM quercetin, 0.2 mM vignacyanidin, and 0.3 mM fatty acid were calculated to be 35.7 ± 10.2, 41.3 ± 2.1, and 42.0 ± 16.0% (*n* = 3), respectively, at 550 nm and 31.3 ± 12.5, 38.0 ± 11.6, and 63.3 ± 15.3% (*n* = 3), respectively, at 700 nm (Figure 8C). The degrees of the suppressions by 0.6 mM quercetin plus 0.2 mM vignacyanidin were 80 and 65% (averages of two experiments) at 550 and 700 nm, respectively.

The effects of flavonoids on pancreatin-induced starch digestion were also studied in the presence of 0.5 mM oleic acid using retrograding joshinko (data not shown). The release of starch fragments from the joshinko particles and the digestion of the released starch were further suppressed by 0.6 mM quercetin, 0.2 mM vignacyanidin, and 0.6 mM quercetin plus 0.2 mM vignacyanidin. 

The above results suggest that (i) fatty acids can effectively suppress the release of amylose, resulting in the preferential digestion of the released amylopectin; (ii) the greater suppression of released starch digestion by flavonoids may be due to the release of amylose and amylopectin, which combined with the flavonoids from the joshinko particles and/or the combining of the released starch fragments with free flavonoids present in the reaction mixtures; and (iii) flavonoids could suppress the starch digestion cooperating with fatty acids.

Starch precipitates prepared using the adzuki extract suppressed starch digestion of the sediment (Figure 7A,B). Then, the effects of the starch precipitates prepared using the above reagents were studied on the starch digestion of the sediment (Figure 7C,D). The precipitates prepared by adding 0.6 mM quercetin plus 0.6 mM oleic acid and 0.6 mM quercetin plus 0.6 mM oleic acid plus 0.2 mM vignacyanidin—which were digested quite slowly compared to the precipitate prepared using the adzuki extract (Figure 7A,B)—suppressed the initial release of starch fragments by about 10% and the digestion of released starch by 30–40% (Figure 7C,D). On the other hand, the precipitate prepared using oleic acid, quercetin, or vignacyanidn was also digested quite slowly but did not significantly affect the digestion of the sediment (data not shown). The results suggest that both amylose/fatty acid complexes and amylose/flavonoid and amylopectin/flavonoid complexes were required for effective suppression under the conditions of this study and that there were other components that could contribute to the suppression of the starch digestion in the adzuki extract.

## 4. Conclusions

The present study indicated that fatty acids, quercetin, and vignacyanidin in the adzuki bean extract could suppress starch digestion in retrograding joshinko by binding to amylose and amylopectin in joshinko particles and that these compounds could also suppress the digestion of fragmented amylose and amylopectin present in the retrograding joshinko by forming complexes with them. It was found that starch fragments combined with fatty acids and flavonoids, which are poor substrates of α-amylase, could suppress the digestion of the starch in joshinko particles and starch fragments released from the particles by surrounding them. According to the findings, it is possible to prepare starch-containing foods using fatty acids and hydrophobic flavonoids, the digestion of which is slow. Although the structure of amylose/fatty acid complexes has been proposed [22], the structures of starch/flavonoid complexes are unclear, and the interactions between amylose/fatty acid complexes and starch/flavonoid complexes are also unclear. Further studies on the structures and the interactions are required to understand how starch/flavonoid complexes become poor substrates for α-amylase and how the digestion of easily digestible starch is suppressed effectively when amylose/fatty acid and starch/flavonoid complexes are added at the same time. 

## Figures and Tables

**Table 1:** Flavonoid concentrations in adzuki extract and molar ratio of each flavonoid in the sediment to the supernatant.

	(+)-Catechin	Taxifolin	Quercetin 7-Glucoside	Rutin	Quercetin	Vignacyanidins
Retention time (min)	13.8	23.1	27.3	29.6	34.7	36.8–42.7 ^a^
Concentration (mM) ^b^	33.5 ± 2.1	26.2 ± 2.7	11.1 ± 0.7	3.2 ± 0.2	36.0 ± 2.1	5.9 ± 0.3 ^c^
Ratio (A/B) ^d^	0.16	0.11	0.26	0.12	0.78	1.83

^a^ Retention times of vignacyanidin and its isomers and derivatives were greater than that of quercetin; ^b^ Concentrations of flavonoids detected in this study. Each data represents mean with SD (*n* = 3); ^c^ The value represents the sum of vignacyanidin, its isomers, and derivatives; ^d^ Molar ratio of each flavonoids in the sediment (A) to the supernatant (B). The data were obtained when 10 μL of adzuki extract was added to 1 mL of joshinko prepared as described in Materials and Methods.
